# What we know about Auditory Charles Bonnet Syndrome?

**DOI:** 10.1192/j.eurpsy.2022.1682

**Published:** 2022-09-01

**Authors:** C. Moreno Menguiano, F. Garcia Sánchez, J.J. Vazquez Vazquez, M.M. Gutiérrez Rodríguez, M.D.L.A. Corral Y Alonso

**Affiliations:** 1 Hospital Universitario de Mostoles, Psychiatry, Mostoles (Madrid), Spain; 2 HOSPITAL UNIVERSITARIO DE MÓSTOLES, Psychiatry, MÓSTOLES (MADRID), Spain; 3 Centro de Salud Mental de Móstoles / Hospital Universitario de Móstoles, Psychiatry, Móstoles, Spain

**Keywords:** old age, Charles-Bonnet Syndrome, auditory

## Abstract

**Introduction:**

Charles Bonnet syndrome (CBS) is defined like visual hallucinations found in individuals who are not necessarily mentally ill, who have visual impairment and no cognitive deficits. Although CBS make reference to visual hallucinations, in this case we are going to deal about Auditory Charles Bonnet Syndrome (aCBS), a very infrequent condition that consists in the presentation of musical hallucinations in patients with sensorineural hearing loss and which etiology is not clearly due to a psychiatric condition.

**Objectives:**

Review the scientific literature available on aCBS to see how much we know about this syndrome.

**Methods:**

Review of available literature sources were obtained through electronic search in PubMed database.

**Results:**

Musical hallucination is a complex form of auditory hallucinations. The most common of these are idiopathic and they present in elderly patients with deafness or impaired audition, which suggests a deterioration of cerebral function. The pathophysiologic mechanism is not understood. These patients tend to have intact reality tests. The time course is variable. In those cases in which it is possible, treating the hearing loss can lead to a significant improvement of the symptom. However, when every this strategies are insufficient, pharmacological treatments can be considered.

**Conclusions:**

- aCBS is an uncommon condition characterized by the presence of complex auditory hallucinations that mainly affect elderly patients with hearing loss. - In most cases there is no previous psychiatric history. - The etiology and pathophysioplogic are not well defined. - There is no etiological treatment. We can use pharmacological and no pharmacological methods of treatment.

**Disclosure:**

No significant relationships.

